# State of Lunacy in the British Asylums

**Published:** 1848-07-01

**Authors:** 


ART. III.—(1.) *The Third Report of the County Lunatic Asylum, at Hanwell, Middlesex, for* 1848.

(2.) *Royal Edinburgh Asylum, for* 1847.

(3.) *The Twenty-Seventh Report of the Dundee Royal Asylum for Lunatics,* 1847.

(4.) *Report of the Medical Officers of the Lunatic Asylum for the County of Lancaster,* 1847.

(5.) *Medical Report to the Managers of the Lunatic Asylum of Aberdeen, for* 1847.

(6.) *Medical Report of the Bedford Lunatic Asylum, for* 1847.

(7.) *Eighth Annual Report of the Crichton Royal Institution for Lunatics, Dumfries,* 1847.

(8.) *Twenty-Eighth Annual Report of the Staffordshire General Lunatic Asylum,* 1847.

(9.) *Report of the Kent Lunatic Asylum for* 1847.

(10.) *Report of the Cumberland Lunatic Asylum, at Dunston Lodge, Gateshead-on-Tyne, for* 1847.

(11.) *The Ninth and Tenth Annual Reports of the Suffolk Lunatic Asylum, for* 1846 *and* 1847.

(12.) *The Royal Hospital of Bethlem: The Physicians' Report.* 1847.

(13.) *The Fifty-first Report of the Friends' Retreat, near York.* 1847.

WE have been anxious to present to our readers a correct idea of the present condition of our principal British county asylums for the treatment of the insane. Much interest has been excited on the subject, not only at home, but abroad, if we are to judge from the numerous letters we have received from parties in different portions of the globe, requesting information in relation to the state of our public institutions for the reception of lunatics. With the view of satisfying our correspondents, we have endeavoured to obtain the most recent reports. Unfortunately, many of the county asylums do not publish any annual reports; others only issue occasional printed statements; so that we find that it will be impossible to lay before our readers a succinct account of the present state of all the public institutions in the country devoted to the reception of insane persons. We have many reports before us, and it will be our object, as far as we possess data, to convey some notion of some of our principal county asylums. The Third Report of the Committee of Visitors of the County Lunatic Asylum, at Hanwell, and the Ninth Report of Dr. Conolly, the visiting physician, first presents itself to our attention. The medical section of the report for 1847

contains little or no information. The former reports have been so full of interesting detail, that we presume Dr. Conolly did not consider it necessary to recapitulate facts or opinions previously published.

On the 31st of December, 1847, there were in the asylum at Hanwell, 971 patients—males, 411; females, 560. Of the males, 189 were daily in the habit of being employed, 187 unemployed, and 37 were returned on the sick lists. Of the female patients, 314 were employed, 209 unemployed, and 37 were under treatment. During the year 1847, 112 persons were employed in the asylum; of these, 18 were officers, 104 servants; of these, 54 were females, and 50 males. The medical officers consist of a visiting physician, who does not board or lodge in the asylum, Dr. Conolly, who receives 315*l.* per annum; of a resident medical officer on the male side, Dr. Begley, who has 200*l.* per annum; and Dr. Hitchman, who has the superintendence of the female side of the asylum, and who receives also 200*l.* a year for his medical services.

The whole of the repairs of the clothing, &c., are done by the patients, with the exception of the repairs of the leather shoes. The washing of the establishment, consisting on the 31st December, 1847, of 1064 persons, is also performed by the patients, with the superintendence and assistance of nine laundresses; and the whole of the cooking, baking, and dairy department, with the superintendence of the housekeeper, and assistance of four servants.

From seventy to eighty patients are generally employed on the farm, garden, and front grounds.

The following is the "Dietary" of the Middlesex Lunatic Asylum. We give it entire, as many of our foreign subscribers have expressed a wish to have this information in detail, in consequence of their inability to procure copies of the Reports of the Asylum:—

### MALES.

#### Breakfast.

Bread, 6 ounces, and 1 pint of Cocoa.

#### Dinner.

| | |
|---|---|
| Sunday, Tuesday, Wednesday, Friday | Meat, 5 ounces, cooked.<br>Yeast Dumpling, 4 ounces.<br>Vegetables, 12 ounces.<br>Beer, half-a-pint. |
| Monday | 1 pint soup.<br>Bread, 6 ounces.<br>Beer, half-a-pint. |
| Thursday | Irish Stew, 12 ounces.<br>Bread, 6 ounces.<br>Beer, half-a pint. |
| Saturday | Pie Crust, 12 ounces.<br>Meat, 1½ ounce.<br>Beer, half-a-pint. |

#### Supper.

Bread, 6 ounces; Cheese, 2 ounces; Beer, half-a pint.

*Extras to Workmen.*

Out-door Workers to be allowed half-a-pint of Beer at 11 o'clock, A.M., and 4 P.M., daily, and 1 ounce of Tea and 4 ounces of Sugar per week.

### FEMALES.

BREAKFAST.

Bread, 5 ounces, and Cocoa, 1 pint.

DINNER.

| Days | Dinner |
|---|---|
| Sunday, Tuesday, Wednesday, Friday | Meat, 5 ounces, cooked.<br>Yeast Dumpling, 4 ounces.<br>Vegetables, 12 ounces.<br>Beer, half-a pint. |
| Monday | 1 pint soup.<br>Bread, 6 ounces.<br>Beer, half-a-pint. |
| Thursday | Irish Stew, 12 ounces.<br>Bread, 5 ounces.<br>Beer, half-a-pint. |
| Saturday | Pie-crust, 12 ounces.<br>Meat, 1½ ounce.<br>Beer, half a-pint. |

TEA.

Bread, 5 ounces; butter, half-an ounce; tea, 1 pint.

*Extras to Laundry-Women, &c.*

Laundry women, &c., to be allowed half-a-pint of beer at 11 o'clock, A.M., with bread and cheese.

N.B.—On Mondays and Thursdays, 11 ounces of currant dumpling is occasionally given in lieu of the soup and stew.

The report drawn up by Dr. Vitre and Mr. S. Gaskell, of the Lunatic Asylum for the county of Lancaster, is extremely satisfactory. They report the steady occupation of the patients, cultivation of their faculties, avoidance of all coercive or harsh measures. This treatment continues to be followed by satisfactory results, inasmuch as it is a means of promoting a more cheerful, contented, and tractable disposition throughout the whole of the patients in the asylum. Amongst a large body of persons deprived of self-control, instances of violence, destructiveness, and attempts at suicide will, under the most judicious management, and in the best constructed building, occasionally occur. The liability to such interruptions to the order, quietude, and well-being of the whole body of persons under treatment is a constant source of anxious solicitude on the part of those whose duty it is to devise and enforce means calculated to prevent such occurrences. Although few or no outbreaks or attempts at self-destruction may arise, yet it is absolutely necessary to observe constant precautions. Continued watchfulness and discretion should be always exercised, even when there may appear little or no danger of accident. During the past year an instance illustrative of these remarks occurred in the asylum: a patient who, by the assumption of a deceptive cheerfulness, was allowed to retire to a dormitory previous to the usual hour of rest, there contrived to commit suicide by means of strangulation. The anxieties and difficulties necessarily connected with such cases are great and not easily surmounted. It is, however, satisfactory to think that among a community of nearly 700 insane persons, many of whom have a strong propensity to acts of this description, only one has effected his purpose during the last five years.

The cultivation of the faculties of the inmates by means of school exercises has for some years formed a valuable auxiliary to the moral system of treatment adopted at Lancaster.

During the year 1847 the establishment contained 663 patients; males, 343; females, 320. Of these, 479 were daily employed; males, 232; females, 247. In the course of the year 66 deaths took place; of these 31 were males and 35 females. At the conclusion of the report a tabular statement is published, showing the proportion of the different classes of insane paupers, not in asylums, but resident in each hundred of the county. Of these there were,

| | |
|---|---|
| Mentally deficient from birth . . . . . | 503 |
| Attacked with insanity . . . . . . . . | 185 |
| Total . . . . | 688 |

The average weekly cost of each patient during the year has been 7*s.* 10$\frac{3}{8}$*d.*

The twenty-seventh Report of the medical officers of the *Dundee Royal Asylum,* drawn up with ability by Drs. Nimmo and Mackintosh, is full of interest. The establishment appears, from the statement in the Report, to be conducted with judgment and skill, the well-being of the patients being the paramount object held in view by the conductors of the asylum. Reference is made in the Report to a remarkable case of remittent insanity.

It is that of a man who, in his best state, is a most amiable being. When his paroxysm is approaching, he will say, "I fear I am about to have a breeze," or "I feel something wrong;" he then submits voluntarily to any remedy, in order, if possible, to prevent its approach. But as the disease gains upon him, he gradually loses all command of himself, an entire change of temper and manner takes place, and at length he is obliged to be secluded, in order to prevent the fearful consequences of his violence. Another peculiar feature of this unfortunate case is, that those he loves, and to whom he is most attached while in his calm state, he hates and abhors above all others while his paroxysm rages.

It appears from the Report that during lucid intervals, some of the patients have a clear and accurate perception of what is right or wrong in all that occurs within the sphere of their own observations. Some time ago, an old and respectable servant of the establishment returned as a visitor, and happened to be ushered into a room where there were two lady patients. One of the ladies immediately assailed her with the most violent abuse, till the poor woman shook again. The other lady, who saw and heard all that passed quite unmoved, after the storm had somewhat abated, calmly spoke thus,—"Such is habit. This servant has not been abused for years. When she was an attendant here, she did not mind this language. Now, I am abused in this manner whenever this lady visits me; but I am habituated to it, therefore I scarcely hear it, and it does not make the slightest impression upon my weak nervous system."

Two of the best working female patients, whose malady has been of long standing, cannot bear to be considered as patients, but insist on being considered as servants. The matron, with her usual tact and humanity, humours this caprice; and the effect is to make them quite easy in their feelings, and even happy in their partial separation from the world.

"The painful feelings of some of our patients, when in their low or depressed state, is so great, that they can scarcely look up; and their cry then is, to get rid of a miserable existence. In this state of mind, everything is a trouble to them. Several of these become indolent and very fat, although indifferent to food, and cold-rife to a very great degree; but when these patients are excited, they care for no person or thing—are utterly regardless of consequences—expose all the faults of their neighbours, especially any moral flaws that may attach to them—lose flesh rapidly—and, in short, become just the reverse of what they were in their low state.

"There is one patient here who has an extraordinarily powerful will. We find the best plan of treatment to adopt with this individual is the 'non-interfering' one, which, we must say, has succeeded admirably, although we have given way in this case to an extent which, were it known, would scarcely be credited."

It appears from the Physician's Report of the *Royal Edinburgh Asylum* for 1847, that during the past year, 251 patients had been admitted into the institution. At the close of 1845, the asylum contained 418 patients; 211 males and 207 females. At the close of last year the patients, both male and female, amounted to 466. The recoveries during 1847 amounted to 98, being in the ratio of 39·04 on the admissions, and of 22 by the mean number resident.

Since the opening of the asylum in 1813, there have been admitted, in all, 1668 patients, and discharged cured, 630; giving an average of cures of 37·76 per cent. on the total number admitted, and, deducting those still under treatment, of 52·2 per cent.

Among the admissions of the past year, there was one of a poor, harmless, little man, who had been chained to the wall of a miserable hut in the Highlands for thirty years, and whose body and limbs were rigidly and permanently bent up into the crouched posture which his bonds had compelled him to maintain so long. He was brought to the asylum in a state of advanced and hopeless imbecility; yet it was in the highest degree gratifying to watch his delight when he found himself once more at liberty, and was daily carried out by an attendant to the open air, where he sat watching the bright rays of the sun.

When speaking of the effects of treatment, the Report says, three cases were admitted, where, in order to subdue excitement, the heroic treatment had been zealously pursued, two of them having been twice bled, and all three otherwise actively and energetically reduced. They were all, upon admission, in an almost hopeless state of fatuity and exhaustion, exhibiting a combination of symptoms closely resembling those of the hydrencephalus of children: one of them sank in spite of every care within a few days after his admission. The second, after infinite care and anxiety, gradually recovered; and the third, after six months of the most complete fatuity, at last indicates the certain prognostics of a gradual restoration to reason.

*Forms assumed by the disease in those admitted.*

| | Males. | Females. | Total. |
|---|---|---|---|
| Mania | 30 | 39 | 69 |
| Puerperal mania | .. | 1 | 1 |
| Mania with epilepsy | 1 | 1 | 2 |
| Melancholia | 18 | 25 | 43 |
| Monomania of suspicion | 17 | 13 | 30 |
| „ pride | 4 | 3 | 7 |
| „ superstition | 3 | 2 | 5 |
| Demonomania | .. | 2 | 2 |
| Dementia | 43 | 25 | 68 |
| „ with epilepsy | 3 | 2 | 5 |
| General paralysis—" Paralysie génerale des alienes" | 7 | .. | 7 |
| Delirium tremens | 2 | .. | 2 |
| Moral insanity | 6 | 4 | 10 |
| Total | 134 | 117 | 251 |

Three of the individuals admitted, voluntarily and spontaneously came to the institution to solicit admission, in order that they might obtain protection from themselves. One of them was a lady who had formerly been an inmate, and who, feeling that an attack of violent excitement was about to seize her, came to obtain shelter against the coming storm. The others laboured under the impulse to self-destruction, and came that they might be watched and preserved from their own hands.

One of the latter cases presented a very singular form of monomania. A young man of high promise, of amiable disposition, superior intellect, and fine moral perceptions, who had pursued, with ardour and success, a long course of classical, literary, and theological study, had been for years haunted by a *single word.* He had long been able to preserve his self-control, and had carried his secret with him in the discharge of his daily duties. But the horrid word was continually before him. Everything suggested it, or led him to fear it would be suggested. It appeared to pursue all his conceptions with the untiring activity and relentless persecution of a demon. It gained upon him every day, until at last it met him in every line he read, and seemed to lurk under every placard, sign-board, and door-plate. Every sound suggested it to his terrified imagination. He could not listen for fear that each word might be the one he so much dreaded; and feared to speak lest it should escape from his own lips. This monad became at last the terror of his existence; and he could no longer trust himself alone, lest he should be impelled to some desperate act, to save himself from his loathsome and inveterate foe.

It appears from the Report that out of 251 patients in the asylum during the past year, 15 have attempted *suicide;* 16 have meditated the act of self-destruction.

"The attempt to commit suicide was exhibited in a paroxysm of maniacal excitement in five; during the progress of general paralysis in one; in three cases of dementia; in eighteen of monomania of fear; and in four of monomania of suspicion.

"In four it had been attempted by drowning; in three, by strangulation; in three, by suspension; in five, by cutting instruments; and in six, by precipitation.

"Of the fifteen by whom the attempt was made, the attempts were single in ten, several or frequent in three, and continued, constant, and unwearied, in two. Several of them made attempts by two or more means, and one by every means conceivable or available."

*Causes of disease in those admitted.*

| | Males. | Females. | Total. |
|---|---|---|---|
| Anxiety | 17 | 9 | 26 |
| Terror | 2 | 3 | 5 |
| Grief | 2 | 13 | 15 |
| Chagrin | .. | 2 | 2 |
| Disappointed affection | 2 | 9 | 11 |
| ,, ambition | 2 | .. | 2 |
| Disappointment | 3 | 3 | 6 |
| Gratified ambition | 1 | .. | 1 |
| Mental fatigue | 5 | .. | 5 |
| Defective education | 1 | 2 | 3 |
| Religious excitement | 7 | 8 | 15 |
| Jealousy | 1 | 1 | 2 |
| Attendance upon an insane brother | .. | 1 | 1 |
| Shock from reading an account of a suicide in a newspaper | 1 | .. | 1 |
| Change of occupation | .. | 1 | 1 |
| Destitution | 1 | 1 | 2 |
| Old age | 1 | 1 | 2 |
| Epilepsy | 7 | 3 | 10 |
| Intemperance | 24 | 6 | 30 |
| General debility | 2 | 3 | 5 |
| Injury of head | 2 | .. | 2 |
| Critical period | .. | 1 | 1 |
| Child bearing | .. | 1 | 1 |
| Amenorrhœa | .. | 3 | 3 |
| Dysmenorrhœa | .. | 2 | 2 |
| Menorrhagia | .. | 1 | 1 |
| Leucorrhœa | .. | 1 | 1 |
| Masturbation | 2 | .. | 2 |
| Dyspepsia | 1 | 1 | 2 |
| Tropical climate | 1 | .. | 1 |
| Abuse of mercury | 1 | .. | 1 |
| Over-exertion in walking | .. | 1 | 1 |
| Fever | 2 | 3 | 5 |
| Unknown | 46 | 37 | 83 |
| Total | 134 | 117 | 251 |

"During the past year, scarcely more than a per centage of 50 to the total cases of mania admitted have been cured, while in former years it has exceeded 70 per cent. On the other hand, a large proportion of cases of Melancholia and Monomania have been discharged cured,—nearly 50 per cent. of the cases of this kind admitted, instead of about 40 as in former years.

"The proportion of cases of Dementia discharged cured is considerable, even when compared with the large number of cases of this kind admitted during the year. It amounts to 16·4 per cent. to the total number of cases of this kind admitted, while the average of the three previous years gives a ratio of only 8 per cent."

When speaking of the mortality during the year, the following interesting case is given:—

It was remarkable for its duration, life having been sustained entirely by the use of the œsophagus tube for a period of more than five months; being, we believe, one of the most protracted cases upon record. The history of the case was also in other respects interesting. The subject of it was a young man of intelligence, industry, and piety, and distinguished by much warmth of affection for his family. A single act of intoxication, and a reproach from his master, led him to throw up his situation: he became dissatisfied with himself, restless, and changeable; and after an incubation of six months, his disease manifested itself distinctly, by alienation of affection for his family, fits of abstraction, ravenous appetite, suspicion, fear, and distrust; and ultimately, after three months in this state, a short time previous to his admission, he was saved from the commission of suicide, by the sudden interposition of his friends. After coming to the asylum, he continued for some time in the same condition; but soon afterwards took to bed,—his pulse fell to 40, his inclination for food appeared to leave him, and all attempts at feeding by the ordinary means were fruitless. He seemed to have lost the sense of taste and the power of deglutition, and lay speechless, motionless, and apparently insensible to all that was done to him, or that went on around. In this state of apparent lethargy, amounting almost to coma, he continued for five months, realizing a worse condition than that which the celebrated John Brown imagined, when he believed that his rational and immortal soul had left him, and nothing remained but the life of the brutes; for here nothing appeared to remain but the mere organic functions of life.

The day before his death, the fire which had smouldered so long, and burned so low, as to escape all observation, suddenly lit up, and he raised himself in bed, spoke, took a basin of broth, and sipped it with apparent relish. On questioning him, it was found, that, during the whole of this period of apparent insensibility, he had been alive to all that had gone on around him,—that he remembered the different attendants who had had charge of him, and appreciated the attentions of each according to their respective merits. It was further discovered that he had refused food, believing that God had commanded him not to eat, and that he could live without food. The most minute objects appeared to have been watched by him, and the operations of a spider, which occupied a corner of the ceiling above his bed, seemed to have been the especial object of his solicitude and observation. A few minutes after he first spoke, he who had so long lived without eating, apostrophised the spider in a tone of commiseration, saying, "Poor thing, it has not had a meal for two days." On the following day he died, and upon a very minute and careful examination of the fauces, pharynx, œsophagus, larynx, trachea, and stomach, no indications of irritation could be discovered. The œsophagus was slightly thickened along a small portion of its anterior wall, near its junction with the stomach, and presented also a small degree of dilatation at this part, which corresponded with the point at which the œsophagus tube must have stopped after its introduction, and where probably its extremity would tend to curve forwards, and thus, by its daily pressure, produce the thickening and dilatation which was observed. The lower lobes of both lungs were studded with tubercles, and contained several cavities of considerable size. The brain appeared to be perfectly natural in every respect.

Of the pathological appearance in the cases in which an examination was made or permitted, twenty-eight in all, the following abstract may suffice. It adds nothing to the experience of previous years. Of the cases examined, two were cases of mania; five, monomania; two, delirium tremens; thirteen, dementia; and four, general paralysis. Opacity of the arachnoid membrane was present in fifteen, of which one was a case of mania, and one of delirium tremens; three were cases of monomania; six, dementia; and four, general paralysis. Sub-arachnoid effusion was present in three—viz., in one of the cases of monomania, and in two of those of dementia. In one of the cases of delirium tremens, the cerebrum presented an anæmic condition; in two of the cases of general paralysis, it was of a violaceous tint; and in all the four it was adherent to the pia mater. In one case, small fibrous tumours were found in the choroid plexus; this was a case of dementia. In eight cases, no morbid appearances were appreciable in the brain—viz., in one of mania; two of monomania; and five of dementia. In several of the cases examined, it appeared that the cortical substance was diminished in depth, but the want of precise information as to the normal thickness of this portion of the cerebral substance, and the difficulty of determining with accuracy its precise depth in any one section that can be made, prevents us, as yet, from pronouncing a positive opinion on this point. The medullary substance was, in several instances, remarkably firm, and of a putty-like or somewhat leathery consistence, being firm, tenacious, and to a certain extent elastic. This was remarked in one case of monomania, but more particularly in several cases of general paralysis, so far confirming the views of M. Foville, that morbid changes in the white substance, consisting mostly in hardening, produced by the adhesion of the planes or fibres of the cerebral substance to each other, are directly connected with disorders in the motive powers.

In one of the cases of general paralysis, in which there was amaurosis, the optic nerves were found very distinctly atrophied along their whole course as far back as their origin in the corpora geniculata.

In one of the fatal cases, in which death was occasioned by peritonitis, it was found that a strangulation of the ileum had taken place by a loop of it having passed beneath a band of condensed cellular tissue, the result, probably, of a previous attack of peritoneal inflammation; sloughing had taken place above the strangulation, with extravasation of the contents of the bowel into the peritoneal sac.

The following illustrations of the power which patients sometimes are capable of manifesting in the concealment of their delusions are interesting:—

"In one, a female of very strong passions, there were a variety of hallucinations both of vision and hearing. People's faces appeared to her to change both their form and colour; she heard voices, and held converse with imaginary forms. Under the influence of an ardent wish to obtain her discharge, she declared that she had got entirely rid of all her false impressions; she even went so far as to explain that a lecture on ventriloquism, which was delivered to the inmates on one occasion, had been the means of explaining to her how she might have been deceived with regard to the fancied sounds. It would have been difficult for a stranger to have discovered in her any trace of insanity; yet, after maintaining her propriety of conduct, and preserving her secret for some time, she suddenly gave way to violent passion on finding that she was not immediately to obtain her liberation; and in the midst of this ebullition, gave full indications that all her hallucinations still maintained their place in her mind.

"The other case was one of still greater interest. It also occurred in a female of amiable dispositions, fond of reading, industrious in her habits, and mild and gentle in her ordinary demeanour. She harboured the illusion that, although in her body and person she was J. A. L., yet that her body was the actual residence of the Divine Spirit which had been incarnate in our Saviour, and was now incarnate in her. With singular inconsistency she wrote a novel, and at all times readily joined in the song or the dance. An attempt was made, by powerful moral agency, to uproot the delusion, and apparently with perfect success. She for a time defended her position with great obstinacy and cleverness, and seemed immovable; but the combined influence of reasoning, ridicule, and addresses made to her other intellectual and moral faculties, at last led her to renounce and repudiate her illusion. She came herself to look upon it with ridicule, and appeared to be completely free from its influence. Some time afterwards, when preparations were being made for her removal, the disappointment of some expectations, which she had been led to entertain regarding the kindness of her friends on leaving the institution, brought back all her former symptoms, combined with others of a similar character; and from her own statement in subsequent conversations, it appeared almost certain that her illusions had never really been dispelled, but were only held in abeyance and concealment for the purpose of gaining esteem, and obtaining her discharge."

In the treatment of insanity, ether and chloroform have both been tried. Of the former agent Dr. Skae says—

"I scarcely require to say anything, as it is already obsolete, farther than to remark that I derived no permanent advantage from its employment. The latter was used by me immediately after the discovery of its anæsthenic agency; and a number of observations were soon afterwards made with it, some of them in the presence of Professors Christison and Simpson. We found that it produced the same physiological effects upon the insane as upon the sane, and that the most violent and excited were almost immediately put into a state of calm and profound repose by its influence. As a curative agent it has as yet been of no benefit in the treatment of the cases in this asylum, although I am not without hopes that, in a certain class of cases, it may be of use. I have, however, found it extremely serviceable for many minor purposes, such as the administration of food by means of the stomach-pump, and of enemata, and in the performance of various necessary operations. I have already recorded my experience regarding its use in our medical periodicals,* and shall only here add one caveat with regard to its employment for the purposes of forced alimentation—namely, that in all probability the loss of sensation which accompanies its use, might greatly mask the ordinary symptoms which would indicate the passage of the œsophagus tube into the air passages; and that without great precaution a fatal accident might happen, which has taken place even in careful hands without chloroform—the injection of the nutriment into the air passages."

On the whole, Dr. Skae's report is ably drawn up, and is replete with useful and interesting information.

It appears from the Tenth Annual Report of the *Suffolk Lunatic Asylum*, for 1844, that the number of patients admitted during the year amounted to 84. The asylum contained, in December, 1846, 329 patients—156 males and 173 females. Thirty-one patients were cured during the past year, 48 died, one was relieved, and one patient escaped. In the house, December 17th, 1847, 248 patients. Dr. Kirkman's report of the asylum is extremely satisfactory.

We have been favoured with the Twenty-Eighth and Twenty-Ninth Annual Reports of the *Staffordshire General Lunatic Asylums*, for 1846 and 1847. We extract the following tabular statement:—

* Monthly Journal of Medical Science, Jan. 1848, p. 544.

| | PRIVATE. | | | PAUPER. | | | TOTAL. | | | |
|---|---|---|---|---|---|---|---|---|---|---|
| | Males. | Females. | Total. | Males. | Females. | Total. | Males. | Females. | Total. | |
| Patients in the Asylum, December 31, 1846 | 30 | 26 | 56 | 112 | 90 | 202 | 142 | 116 | 258 | 317 |
| Admitted during the year 1847 | 3 | 12 | 15 | 25 | 19 | 44 | 28 | 31 | 59 | |
| Discharged recovered | 1 | 8 | 9 | 11 | 10 | 21 | 12 | 18 | 30 | 61 |
| „ relieved | 1 | 4 | 5 | 1 | 1 | 2 | 2 | 5 | 7 | |
| „ as incurable, or at request of friends | .. | .. | .. | 3 | 4 | 7 | 3 | 4 | 7 | |
| Died | 2 | 2 | 4 | 8 | 5 | 13 | 10 | 7 | 17 | |
| Remaining in the Asylum, December 31, 1847 | 29 | 24 | 53 | 114 | 89 | 203 | 143 | 113 | | 256 |
| State as to probability of recovery: Curable | 3 | 4 | 7 | 8 | 6 | 14 | 11 | 10 | 21 | |
| State as to probability of recovery: Incurable | 26 | 20 | 46 | 106 | 83 | 189 | 132 | 103 | 235 | |
| Average number resident throughout the year | .. | .. | 54 | .. | .. | 204 | .. | .. | 258 | |

*Return of Patients admitted on or discharged from the Second Class, or Charitable Subscription Fund.*

| | | |
|---|---|---|
| Remaining on the Books, December 31, 1846 | 30 | 36 |
| Since admitted | 6 | |
| Discharged recovered | 5 | 7 |
| Died | 2 | |
| Remaining in the House, December 31, 1847 | | 29 |

### STATISTICAL TABLES.

| FORM OF THE DISORDER IN 59 CASES ADMITTED IN 1847. | Males. | Females. | Total. |
|---|---|---|---|
| Mania { Acute | 8 | 5 | 13 |
| Mania { Ordinary | 7 | 11 | 18 |
| Monomania | 2 | .. | 2 |
| Melancholia | 3 | 8 | 11 |
| ,, with suicidal disposition | .. | 2 | 2 |
| Moral insanity | 1 | 1 | 2 |
| Imbecility and dementia | 4 | 4 | 8 |
| Idiocy | 1 | .. | 1 |
| Epilepsy | 2 | .. | 2 |
| | 28 | 31 | 59 |
| **DURATION OF THE DISORDER ON ADMISSION.** | | | |
| Not exceeding 1 month | 6 | 4 | 10 |
| ,, 2 months | 7 | 5 | 12 |
| ,, 3 months | 2 | 4 | 6 |
| ,, 6 months | 3 | 3 | 6 |
| ,, 1 year | 2 | 7 | 9 |
| ,, 2 years | 3 | 5 | 8 |
| More than 2 years | 5 | 3 | 8 |
| | 28 | 31 | 59 |
| Cases of first attack | 14 | 11 | 25 |
| Cases of more than one attack | 9 | 13 | 22 |
| Not ascertained | 5 | 7 | 12 |
| | 28 | 31 | 59 |
| **CIVIL AND RELIGIOUS CONDITION, &c.** | | | |
| Married | 15 | 11 | 26 |
| Single | 13 | 15 | 28 |
| Widowed | .. | 5 | 5 |
| Established Church | 14 | 17 | 31 |
| Roman Catholic | 1 | 4 | 5 |
| Dissenters of various denominations | 7 | 5 | 12 |
| Doubtful and unknown | 6 | 5 | 11 |
| Of fair education | 4 | 5 | 9 |
| Able to read and write | 8 | 7 | 15 |
| Able to read only | 6 | 11 | 17 |
| Totally uneducated | 10 | 8 | 18 |

STATISTICAL TABLES—*continued.*

| AGE OF PATIENTS ON ADMISSION. | Males. | Females. | Total. |
|---|---|---|---|
| From 15 to 20 years | 2 | 1 | 3 |
| „ 20 to 25 „ | 5 | .. | 5 |
| „ 25 to 30 „ | 1 | 7 | 8 |
| „ 30 to 35 „ | 4 | 4 | 8 |
| „ 35 to 40 „ | 2 | 4 | 6 |
| „ 40 to 45 „ | 5 | 3 | 8 |
| „ 45 to 50 „ | 5 | 2 | 7 |
| „ 50 to 55 „ | 2 | 3 | 5 |
| „ 55 to 60 „ | 2 | 3 | 5 |
| „ 60 to 65 „ | .. | 3 | 3 |
| Above 70 | .. | 1 | 1 |
| | 28 | 31 | 59 |
| PROBABLE CAUSE OF THE DISORDER. | | | |
| Anxiety | 1 | 7 | 8 |
| Congenital defect | 2 | .. | 2 |
| Disease of the brain | 3 | 1 | 4 |
| Disappointed affection | .. | 3 | 3 |
| Epilepsy | 2 | .. | 2 |
| Fever | .. | 1 | 1 |
| Intemperance | 10 | 2 | 12 |
| Injury to the head | 1 | .. | 1 |
| Puerperal and uterine disorders | .. | 3 | 3 |
| Religious excitement | 2 | 1 | 3 |
| Reverses | 2 | .. | 2 |
| Unknown | 5 | 13 | 18 |
| | 28 | 31 | 59 |
| Hereditary disposition to the disorder ascertained in | 6 | 7 | 13 |
| CAUSES OF DEATH IN 1847. | | | |
| Accidental choking | 1 | .. | 1 |
| Bronchitis and age | 1 | 1 | 2 |
| Disease of the brain | 3 | .. | 3 |
| Epilepsy | 1 | .. | 1 |
| Gastro-enteritis | 1 | .. | 1 |
| General exhaustion | 1 | 3 | 4 |
| General paralysis | 1 | 1 | 2 |
| Phthisis | .. | 2 | 2 |
| Valvular disease of the heart | 1 | .. | 1 |
| 3 died in January, 2 in February, 4 in March, 2 in April, 2 in May, 3 in June, and 1 in October. | 10 | 7 | 17 |

*General Statement of Patients admitted, discharged, and now on the books, from the opening of the Asylum, Oct. 1st,* 1818, *to Dec. 31st,* 1847.

| | | |
|---|---|---|
| Total number of admissions | | 3307 |
| Discharged recovered | 1438 | 3051 |
| „ relieved | 461 | |
| Removed, as harmless or incurable, or by desire of friends | 471 | |
| Died | 681 | |
| Remaining under cure | 21 | 256 |
| „ incurable | 235 | |

The Medical Report of the *Lunatic Asylum of Aberdeen*, for 1847, contains many interesting particulars. It is drawn up by Drs. Macrobin and Ogilvie. It appears from the Report that the—

"Admissions have been 67 in number—viz., 31 males and 36 females, while there remained in the house at the commencement of the year 208, so that in all 275 have passed under treatment; of whom, 29 have left the institution recovered, and 18 more or less improved; three have been removed without material improvement, or have been dismissed as improper objects, and 12 have died; leaving at the end of the year 213, of whom 116 were males and 97 females.

"The mean number of patients for the entire year has been 213·48—viz., 114·47 for the males, and 99·01 for the females. To obtain these averages as accurately as possible, an entry was made in a record kept for the purpose, on occasion of each admission removal and death, of the number of patients residing in the house; and at the end of the year these numbers were added up and a general average taken.

"It will be seen from Table II. that the districts which continue to receive most benefit from the institution are the town parishes, and those rural parishes lying within the limits of the county of Aberdeen, especially the former, though the number of inmates belonging to this class does not preponderate so decidedly over those included in each of the other two in the table as it did last year.

"A table shows the ages of the patients admitted. It exhibits, as usual, a great preponderance in the number of cases between the ages of 20 and 50, amounting as they do to about 85 per cent. of the whole number. The only one under the age of 20 years was a boy of 13 labouring under recurrent mania, and who, we believe, is the youngest patient ever received into the institution, with the exception of one, a boy eight years old, who was admitted in 1839, and recovered after a short residence.

"In 46 per cent. the form assumed was mania; in 24 per cent. monomania; in 16½ per cent. melancholia; in 9 per cent. dementia; and in 4½ per cent. moral insanity."

In reference to the origin of insanity, the Report says—

"In a considerable proportion of the admissions, intemperance in the use of intoxicating liquors has had a share in inducing the complaint; and in twelve of these—of which ten were males and two females—we have reason to believe that it was the principal agent. Two out of the ten were complicated with general paralysis, of which the one died a few weeks only after admission, while the other we fear is likely soon to follow.

"In addition to the three cases enumerated in the table under the head of mental anxiety as a primary cause, it appears to have acted in three or four others as a secondary one, but in none of them, of which a satisfactory history has been obtained, does it appear to have stood alone, and we believe that it is not often found to do so. It is when this cause co-exists with intemperance or other habits which tend to impair the vigour of the nervous system, with hereditary predisposition, or with overstrained mental exertion, that it is most certain of producing its effects.

"One, if not two instances, have occurred in which the excessive use of tea is thought to have injured the constitution, and so become indirectly a cause of the malady; and there appears to be reason for supposing that this is not a very uncommon occurrence among females in the lower ranks of life, who frequently use that luxury to an immoderate extent, and to the exclusion of more nourishing articles of food.

"In one of the cases above alluded to, that of a female, the insanity is said to have been more immediately induced by religious excitement, a cause which acts most strongly on that sex, but one of which the operation has, we think, been frequently misunderstood, and the effects over-rated. While excitement on topics connected with religion has sometimes unfortunately been productive of injurious consequences on minds not naturally strong, it has been remarked by a high medical authority, that systematic religious training and truly devotional feelings, established as a habit of the mind, form, in fact, its best safeguard.

"Where poverty has acted as a cause, it appears to have done so principally from the physical hardships and privations attendant on it, but something seems also due to the reckless and desperate feeling it is apt to generate in ill-regulated minds.

"The unmarried are found, as usual, to be more numerous than the married or widowed.

"Hereditary predisposition and previous attacks include each about 30 per cent. of the whole number, and are found, as usual, to co-exist, in a good many instances, in the same individual. In one case which falls under the latter head, repeated attacks of delirium tremens have been held equivalent to the previous occurrence of insanity, usually so called. Paralysis had occurred in three, including the two cases of general paralysis already alluded to; the third being, it is understood, the result of arsenical poisoning, although it closely simulated the malady in question. Two male patients—an unusually small proportion—were epileptics. Hysteria, illusions of the senses, and insane impulses, were also present in several instances. Scrofula, a common affection in this part of the country, and phthisis, were each of them present in one of the cases admitted.

"The recoveries have been twenty-nine in number—viz., ten males and nineteen females, being 43·28 per cent. to the admissions, and 13·58 per cent. to the mean number resident. As regards the two sexes separately considered, these proportions are 32·26 and 8·74 respectively for the males, and 52·78 and 19·19 for the females, the balance being thus considerably in favour of the latter, on whichever of the numbers the per centage is calculated—a result which confirms, in so far as last year's experience goes, the remark made in a former report—namely, that though the female patients in the house, at any given period throughout the year, have been less numerous than the males, a greater number pass under treatment at the same time, a greater number recover at the end of the year, and, it is to be feared also, that a proportionably greater number afterwards relapse. The average term of residence has been rather more than ten months, (43·83 weeks.)

"The deaths have been twelve in number—viz., seven males and five females; being for the former, 22.58 per cent. on the admissions, and 6·11 per cent. on the mean number resident; for the latter, 13·33 per cent., and 5·05 per cent.; and for the entire number, 17·91 per cent., and 5·62 per cent. respectively; thus tending to confirm the opinion founded on the experience of this and most other asylums in regard to the greater mortality of male lunatics. Two of the deaths were the result of general paralysis, two of apoplexy, one of epilepsy, and one of pulmonary consumption—all frequent complications; two of debility, also a common cause of death among the insane; and three of old age and exhaustion—a source from which we must expect an annually increasing mortality, as the number of aged inmates in the institution continues to augment. The remaining death, which was due to peritonitis, affords a striking illustration of the difficulties the physician has to encounter in treating incidental maladies among the insane. The patient, who was affected with hemiplegia, and who was besides quite fatuous, was unable to describe her sensations, and the pulse was but slightly affected, so that the symptoms, usually most unmistakeable in this malady, afforded very insufficient means for forming a diagnosis. On examination after death, an ulcerated opening was detected in the coats of the stomach, through which the contents of that viscus had been effused into the peritoneal cavity."

The Report in question contains some very valuable tables; among them we would direct the particular attention of our readers to the one exhibiting the pathological appearances discovered after death in those who were made the subject of post-mortem examinations.

The eighth Annual Report of the *Crichton Royal Institution for Lunatics* (*Dumfries*) is now before us. This institution, which takes a very high position among the asylums in that part of the British empire, is under the able superintendence of Dr. A. F. Browne, who is the resident physician of the establishment. The Reports annually issued by the medical officers of this asylum, are more than ordinarily interesting and valuable. Much pains appear to be taken in drawing up the medical statement, the statistical data being interspersed with practical suggestions for the general medical and moral treatment of the insane. This establishment contains 207 patients. Thirty were discharged cured during the year 1846, nine were said to be relieved, and fourteen died. In speaking of the fatal cases, Dr. Browne says, in three

"There was observed that return to intelligence in the last hours of life which was formerly supposed to be invariable in insanity, and which is so ardently desired by friends and relatives. Either from the altered state of the circulation, the effect of suffering, the incompatibility of two morbid actions, or the counter-irritation produced by distant disease, the brain sometimes, as in these examples, partially recovers its normal functions, and the incoherent speak rationally and collectedly, the violent become calm, and the clouds are dispelled which have previously darkened the memory. In one of these patients, resignation and recollection replaced irritability and imbecility; in two others, the dispositions were softened and elevated, although the characteristic delusions remained. In two were observed that resistance to the influence of mental disease, and insensibility to pain, which have most erroneously been asserted to constitute diagnostic symptoms of insanity; but which prevail to as great an extent in phrenitis, fever, and intoxication. A lady seemed to be altogether unconscious of suffering, and continued to smile and sing while external circumstances indicated the pressure of excruciating agony; and a man walked about labouring under severe broncho-pneumonia until within two days of his death, and until the disease was detected, not by his statements, for he conceived that he possessed the most robust health, but by the stethoscope."

In speaking of "derangement by impairment," Dr. Browne observes—

"It is an inquiry of some importance at what period, and under what circumstances, the human mind naturally becomes impaired—shorn of its splendour, or deprived of its strength or characteristic attributes. Every constitutional change, every grave disease, every source of disquietude inflicts, or may inflict, injury, may lessen the aptitude to receive or retain impressions; the power to compare, separate, combine its own acts; the capacity for sorrow or for joy. It is only when these privations are multiplied and magnified, so as to obtrude upon the interests or sympathies of others, that the evil is recognised and treated; but the consciousness of the majority of reflecting men, who have arrived at maturity, affords evidence of the effects of time, and toil, and excitement; of the enfeeblement of particular powers, the extinction of a passion, or the loss of portions of knowledge. Impairment of mind is the most common form of derangement in asylums. Of sixty-one cases in the past year, twenty-four presented this symptom. Of this number six voluntarily placed themselves in the asylum, affording a commentary upon the modern treatment of insanity, and illustrating the entire confidence of many of the insane in the humanity and expediency of the provisions for their care and cure. The rudimentary stages of the affection generally occur in the world, in the conflict and confusion of active life, amid the turmoil to which they may owe their origin. When isolation is resorted to, the injury is conspicuous, the wound ghastly and gaping; and that process, which commenced in the forgetfulness of a single word, or class of words, is found to extend to a whole language; or that indifference to the kindness of one friend, which was at first attributed to caprice, is presented as hebetude and callousness to every social and domestic affection."

The particulars of a case of insanity in a deaf mute are recorded, as presenting in its progress some interesting psychological phenomena. They were—

"1. That he wrote his delusion as to his capability of speaking in the same imperfect and incomplete manner that paralytics do. 2. That he spoke incoherently on his fingers. And 3. That he lost the knowledge of the digital alphabet gradually, recollecting a few of the signs, such as S and H, much longer than others, and repeating them incessantly in his vain endeavours to render himself understood."

Dr. Browne refers to a numerous class of cases of mental impairment, (which is often to be seen both *in* and *out* of asylums,) in which the morbid condition of mind is exhibited in a perversion of the controlling faculty—the helm of the understanding. He says—

"These persons are facile, irresolute, incapable of a continued pursuit; they require to be protected from their own vacillation, to be moved by the will of others; they are comparatively sane under the shelter of an asylum; but lapse into puerility and pusillanimity when called upon for exertion or cast upon their own resources. Their condition is the fruit of an education which, while it imparted the refinement of accomplishments, withheld that training which is the basis of all moral and mental health and stability. In four cases, there is presented that impairment of personal courage, and of the power of analyzing or excluding our own sensations, and of recognising our true position, which enter so largely into hypochondriasis. One of these involuntary cowards was convinced that he could neither sleep, walk, nor think: another that he was ignorant of the principles of his trade, and unable to conduct business: and a third that he is about to die—that his heart has ceased to beat. In one lady there appears to be a total incompetency to conduct the ordinary affairs of life—to regulate a household, educate her children, or act in accordance with the plausible doctrines which she delights to inculcate: in another there is a signal absence of truthfulness—a pretension to sentiments of piety and purity, which she does not feel—a tendency to act a lie: in a third, with general feebleness and frivolity of character, there are very obscure notions of the rights of property, and a tendency to appropriate, and hoard, and conceal articles to which she has no claim, of which she can make no use, and which tend rather to incommode her personal comfort."

These are sad cases; they deceive the inexperienced; it being difficult to persuade persons who have had few opportunities of becoming practically acquainted with the many phases of mental malady that this is the most lamentable, and often the most dangerous, form that insanity can assume. These cases are the least to be trusted; they frequently require to be kept under surveillance for a considerable period, occasionally during a term of life. In fact, when the moral faculties are morbidly vitiated, more danger is to be expected than would result if the impairment was confined principally to the powers of ratiocination. Care must be taken to draw a philosophic distinction between a *natural* and a *diseased* vitiation of the moral faculties.

Dr. Browne refers to a case of "temporary fatuity" manifesting the following symptoms:—

"A man of active temperament suddenly becomes irritable, then lethargic, and ultimately stupid and imbecile; seems lost to all external impressions, and unconscious of internal motive or impulse, utters a monosyllable, or ceases to speak, and is deaf and dead to all appeals, whether made to his sense of fear, or shame, or vanity, or honour. The restoration of his former powers is generally rapid and complete. But, for a season, this individual is as essentially isolated and as incapable of removing the barrier which separates him from his fellow-men as when the disease is permanent."

Dr. Browne says this form of insanity can generally be traced to structural disease of the brain. We doubt it. As far as our experience enables us to form an opinion, we have reason to consider that this condition of mind is the result of some disturbance of the circulation in the brain, unconnected with what is understood by the term of "structural disease." This disturbance of circulation is often the consequence of irritation or disorder remote from the centre of nervous vitality. It is frequently the cause of what is called "intermittent insanity," which is generally curable by a perseverance in the use of means to restore and sustain a healthy balance of the cerebral circulation. In many of these cases, the *liver* is the primary seat of the malady. We are speaking of the cases to which Dr. Browne refers, as well as to that form of insanity which is decidedly intermittent in its character. Dr. Browne maintains what we have always advocated, that in the insane consciousness may be clear and exact; the patient may be perfectly cognisant of all that is said or done by himself, and of the tendencies and effects of what is said or done; and this knowledge may not only exist at the moment, but be retained by memory, and serve to regulate subsequent conduct, but fail altogether to influence the morbid manifestations: or, secondly, consciousness may be natural during a paroxysm or period of excitement, become obscured, imperfect, or obliterated on the subsidence of the agitation, but return when a relapse takes place, preserving the same vividness and exactitude which it originally possessed, reproducing, after a long interval, passed in total ignorance or forgetfulness of these ideas, the most minute events which took place during the previous attack, the same modes of expression, and the same state of emotion, phenomena which have been supposed to countenance the theory of a duality of being; or, thirdly, consciousness may be at all times impaired; the patient does not observe, does not feel, attend to, obey, or retain the impressions conveyed to him, nor the processes of his own mind. The possession of consciousness in many of these cases is one powerful argument in favour of the kind and humane treatment of the insane. But need we, in the present day, advance such an argument in such a cause? All our best sympathies ought to be on the side of the insane, irrespectively of the presence or complete loss of consciousness.

We find in the report the following just observations on the subject of *suicide* :—

Suicide is at present epidemic in France. It has been, and again threatens to be, epidemic in this country. When this is the case, the deed is perpetrated either from imitation, from the suggestive instinct, or it is the result of circumstances affecting the whole community, and acting upon minds similarly constituted, and similarly disorganized. During political convulsions, panics, plagues, famines, the number of suicides is usually great. The same causes which increase the general mortality may diminish the value or love of life in the individual. At such a crisis, the self-destroyer dies to escape from the terrors of death. Then there is less a disgust at life than an anticipation of incompetency to discharge the duties and calls imposed upon industry, or courage, or virtue. Where doubt, or despair, or terror, assail the mind, death may be sought under the instigation of a sudden impulse, but, in the great majority of cases, it is a deliberate act, the result of a perverted or corrupted moral sense, it is true, but contemplated as the possible close of life, or of certain circumstances or course of conduct for which preparations have been made, and the means purchased and preserved for years. A gambler rises from hazard, and at once blows out his brains; a minister of state quails under the storm of popular indignation, and swallows arsenic; but inquiry will disclose that the pistol and poison have been treasured, and perhaps labelled, as friends in an emergency. But, where neither adequate cause, nor motive, nor object, can be recognised, suicides occur from, or may be legitimately referred to, a mere morbid tendency—a loss of the desire to live, and think, and enjoy—a disposition to destroy the consciousness of pain. The impulse is as uncontrollable as that which leads to homicide. It may be regarded, and reasoned upon, as an infatuation by the patient, but it cannot be excluded from the mind, or subdued; it is dreaded, defied, repudiated as a principle of action, and resisted as repugnant to the principles of the actor, but it conquers. However worthy of being classed among mental diseases, when emanating from a motive real or imaginary, it is chiefly so when it exists as a perversion of a moral attribute, and it is then chiefly dangerous and intractable. The furious and frantic rarely attempt, although they may threaten, to commit suicide. It is the calm, rational, melancholic, who resists all treatment, repels all counsel, who at last effects his purpose, despite of all vigilance. It is likewise formidable when found in conjunction with a strong understanding, where its nature and consequences can be estimated; and as a concomitant of fatuity and senile insanity, where the mind is a blank or in total confusion, and incapable of appreciating its own condition, or of acting under a settled purpose. Patients more frequently commit suicide when recently admitted to an asylum, and when about to return, or when they have actually returned home, than at other times, as the tendency is obviously most irresistible at the invasion of the disease, and during convalescence; when the impulse is newly developed, and when the removal of restraint, the renewal of former associations and intercourse, expose the nervous system to new and powerful excitement. In seventeen cases of monomania with delusion, there have been six presenting the disposition to suicide as the most prominent symptom. In four of these the mental eondition was simply a desire to die—to flee from some one state or object to another : in two the desire originated in, and was traced by the patients themselves to remorse—to a conviction that, in ceasing to live, they might cease to suffer. Fashion, or imitation, or some new invention, generally determines the mode of destruction, as at present many suicides prefer death by prussic acid, or by the sweep of a railway train; but in the cases under review, all attempted or meditated death by strangulation.

In speaking of the *classes exposed to insanity*, Dr. Browne observes: —It has been affirmed that the classes from which asylums are peopled, especially with cases of excitement and perversion, can readily be pointed out in society, and that, with a moderate degree of penetration, the ultimate mental condition—the fate of particular individuals and families may be foreseen and foretold. These are tendencies, and habits, and pursuits, which lead directly to mental disturbance and unsoundness; but there are likewise qualities and dispositions which, of themselves innocuous, are gradually developed, and exalted, and matured into extravagance, eccentricity, and disease: there is an education provided in many of our social arrangements which promotes and precipitates the process: there is a moral atmosphere created by the prevailing tone in religion, politics, and commercial enterprise, which may act favourably upon those of strong and stern temperament, or may not act at all, but proves fatal to the fragile and susceptible. The Hercules in frame and intelligence may fall beneath the blows of misfortune, or the crash of a panic; but imaginative and excitable minds are affected and wrecked by the influence of ordinary circumstances, by the very training to which they have been subjected, the course which they have pursued, and the destiny which they have courted. If the history of the insane be traced, where no specific cause of the disease has been or can be assigned, there will be generally found some peculiarity of thought or conduct which has issued in eccentricity; some acute susceptibility of feeling which, by indulgence and cultivation, terminates in melancholy; some infirmity of purpose, which becomes incapacity; some irritability of temper, which expands into violence; some vain, but cherished opinion, which eventually monopolises the whole mind; some secret, forbidden taste; some hidden spring of hope, or ambition, or fear, which may be a foible in youth, but is regarded as folly in manhood—which may be compatible with prosperity, but is laid bare, and exposed as visionary and preposterous under misfortune. If imbecility is often indicated by such minute irregularities as absence of mind, reverie, abstraction, forgetfulness of facts or persons, so are other forms of alienation preceded by mere gentleness and feebleness of character, by slight inconsistencies, or contrarieties of disposition, or even oddity in dress. While it may be held as certain, that physical disease is the cause or contemporaneous condition in every case of aberration, a number of patients have been admitted recently whose previous history presents little for observation, except some pre-existing or premonishing peculiarity or perversity."

Dr. Browne refers to the subject of the simulation of disease among the insane. He considers "malingering" to be a symptom of insanity. The case of a lady is reported, who alleged that she spat blood in enormous quantities, and endeavoured to produce practical proof of the hæmorrhage. A gentleman, discharged from the asylum, acted paralysis with great success; rheumatism, neuralgia, toothache, are of constant occurrence; pain of every description, in every position, is complained of; epilepsy is so well simulated that the pretended orgasm produces a modified condition, but closely allied to the true paroxysm. This tendency is most frequently observed among females. Its most exquisite form is likewise seen in that sex. An example of long-sustained and successful voluntary deception of this kind lately occurred in the establishment. A lady, of elegant manners, and good education, appeared to be plunged in profound lethargy; to be mute, insensible to external impressions, even of hunger. She refused food; she resisted its administration, and was compulsorily fed for nine months; she never moved, except when impelled; her head reclined upon her bosom; her eyelids were firmly closed; her limbs were cold and motionless. Every means were resorted to, first, to remove whatever bodily condition might have provoked or prolonged these symptoms; secondly, by solicitations, admonitions, threats—by exercise, music, amusement, and other moral excitants, to shake the obduracy of the will; and, thirdly, by the use of external means, calculated to rouse the nervous and muscular systems, and remove this stupor. But this individual, who seemed utterly callous to such irritants, who seemed indifferent to the shower-bath, or who shrank under the shock for a moment only, and who remained firm and inflexible under the action of electro-galvanism, suddenly, and through the influence of a powerful motive, raised her head, looked around, spoke loudly and intelligibly, walked with ease, took her food, displayed solicitude as to her property, and, although still insane, has for upwards of a month presented no indication of torpor, or taciturnity.

It is highly consolatory to hear a man of Dr. Browne's experience state, when speaking of the "curability of insanity," that from 30 to 40 per cent. of the insane, whatever may be the species, or extent, or duration of the disease under which they labour, are restored to reason during confinement or treatment, or to such an amount of self-control and intelligence as to resume their place in society, and the exercise of functions and trades involving great trust and responsibility. It may, in fact, be contended, that, while the obstinate nature of the disease is admitted, it is as curable as other affections of equal magnitude. The force of this argument will be seen when it is stated that the cases treated and relieved in private are not known, that it is only the chronic, and unmanageable, and the desperate cases which are sent to asylums—which are thus converted into hospitals for incurables. It must further be recollected that insanity is not one disease, but a group of diseases, of which many species are, from their nature and complications, incurable, and ultimately fatal. If these be deducted from the numbers under treatment, upon the same principle that incurable cases are excluded from hospitals for other diseases, and an estimate then formed of the results, the conclusion will not be discouraging. It is true that great excitability and eccentricity sometimes characterise those who have been subject to alienation; but it is believed that the condition of the brain in such cases is as sound and subservient to the discharge of its functions as that of the lungs which have been subject to pneumonia, or of the tissues which are liable to gout. However limited the powers of art may be in removing such an affection, it is very evident that, in proportion to the failure of the attempts to cure, ought to be the provisions for the care of the insane; and that, although the rapid spread of the disease may not portend the realization of the extraordinary opinion of Bishop Butler, that whole communities may, under certain circumstances, become mad, it is now absolutely necessary to extend the means of secluding the insane, and of protecting the public from the evils which arise from contact with debased appetites, violent passions, and excited imaginations.

The following practical and judicious observations with respect to the administration of medicine to the insane, are deserving of attentive consideration. Upon this field the battle is often to be fought and the victory to be obtained. Having great faith in the power of medicine in restoring the mind to its healthy and normal condition in a large class of insane patients, it becomes a question of great importance, how is a consecutive plan of medical treatment to be carried into operation? How difficult it often is to induce patients to take any quantity of medicine! Dr. Browne says—

"It is a noble, and prudent, and humane principle to avoid all deception in the management of the insane; to prefer force to fraud. This holds true in everything where force can succeed, where it is not dangerous, where it does not defeat the object in view; it especially holds true in the exhibition of medicine, as, when placed surreptitiously in the food, or disguised, it may lead to the suspicion that the meals are poisoned, medicated, or unwholesome, and that they are always so. But it would be absurd to assert that we must not have recourse to means to conceal the employment of medicine. It must be mixed with the food, and in such a way as to elude the senses, and the ever vigilant suspicions, of the patient, aroused, as these will necessarily be, by the entreaties to take voluntarily and openly, what is subsequently offered under false pretences. Medicine is required in many cases, especially laxative and alterative drugs, every day; and if there be grave objections to the forcible administrations of food, these apply with greater stringency to the forcible administration of mixtures and powders. The apparatus, the determination of the operators, all countenance the horrible idea that murder is about to be committed. The opinion that one resort to force generally suffices to overcome the obstinacy or fears of the recusant is altogether fallacious. There are patients in the Institution who have been compulsorily fed three times a day for six months. Abstinence often depends upon gastric irritation or constipation, and can only be overcome by removal of the cause. Croton oil is recommended, and used, as a ready means of relieving the most prominent symptom, and of triumphing over the perversity of the individual at the expense of little strife and resistance, as it acts if placed upon the tongue; but it is unsafe to employ so powerful and drastic medicine in the great majority of cases of gastric irritation, and it is equally unwise to run the risk of a struggle during acute disease. From these and other considerations, and subsequent to a fair trial of reasoning and persuasion, medicine must be exhibited under various disguises. Gin is a good diuretic in cases of chronic dropsy, and is always acceptable, even when combined with others. Lozenges are in constant demand. They may be called the current coin, the peace-offerings of such an establishment. Under this form, calomel, ipecacuan, magnesia, morphia, gelatin, nitrate of potass, may be given in small doses. Castor oil may be converted into a custard; croton oil may escape notice in the centre of a slice of bread; calomel may be spread with the butter. Scammony is not distinguished from pepper in broth; and gamboge emulates the colour of eggs in pudding. The appetite may be pampered, and a favourite dish may be used as the vehicle of what, under other circumstances, would inevitably be rejected. Porter may be resorted to as a tonic, and bitter, and astringent, and the soluent of colocynth, aloes, quassia, sarsaparilla, or catechu."

The *Dunston Lodge Lunatic Asylum,* in the county of Cumberland, under the medical care of Drs. D. B. White, and C. Lockhart Robertson,* has been in existence for sixteen years; during that time, according to the Report for 1847, now before us, 584 patients have been admitted; of these 328 were males and 256 females. Discharged, recovered and improved during that period, 362 : 203 male, and 159 female patients. Died, 97 : 60 males, and 37 females. Remaining in the institution on the 1st of January, 1847, 125 patients : 65 males, and 60 females. From the tabular statements the following conclusions have been drawn :—

* This gentleman is now associated with the Military Lunatic Asylum at Yarmouth.

"1stly, That, on the total admissions for the sixteen years, 362, or 61·9 per cent., have been discharged more or less benefited by treatment.

"2ndly, That, on the total admissions for the past year (1846), 49, or 77·7 per cent., have been similarly discharged.

"3rdly, That, on the total of the Cumberland paupers admitted during the same year (1846), 23, or 65·6 per cent., have been sent home more or less improved."

The table in question establishes the advantage of early removal to an asylum ere the fatal progress of unchecked or mistreated insanity has beclouded the mind beyond the reach of art. 31·4 per cent. of the 65·6 per cent. discharged as above, were cases admitted during the first stage of the disease, to the effecting of whose cure a *few months* residence only was required, while the average residence extends over a period of *two years*.

The following fact proves that the humane and Christian system of treating the insane is not universally followed. The Report says—

"Even within the present century, the moral treatment of the insane consisted generally in chains and stripes. The body bound with iron, the mind enslaved by fear, was the state man doomed his fellow-man to, striving, as it were, to add his vengeance to Heaven's visitation. Even now—hear, Oh, Christian England—are similar barbarities practised. On the 26th of November last, a pauper lunatic was brought to this asylum, his arms and legs fettered; the irons of each extremity connected by a massive chain; and long use had so rusted the screws, that, at the risk of injury to the limbs, it was necessary, for the purpose of effecting the poor man's liberation, to cut the irons with a chisel. The knee-joints were found so contracted that he could neither walk nor stand upright. His mental powers were entirely destroyed. Memory, nay, the power of comprehension, being lost.

"From his history, it would appear that his *two sisters*, with the view of retaining the command of some property belonging to him, had kept him in an outhouse chained hand and foot, and fastened to a staple in the floor. In this horrible situation, naked, save a few canvas rags, with no other bed than a little straw and a few leaves, and compelled by the shortness of his chain to remain constantly in a crouching attitude, had he for twelve years continued, when rumours of his condition at length attracted the notice of the magistrates of the district, who, on ascertaining their correctness, at once ordered his removal to an asylum."

In order to show the impunity with which lunatics may be intrusted with various agricultural instruments, the Report says—"So many as thirty-three of the patients were employed last harvest in the reaping field without any untoward accident. It would seem as if the confidence reposed in them begets a like confiding temper; cheerfulness and peace replacing discontent and clamour—a result thrice counterbalancing the risk. In like manner have, during the year, joiners and shoemakers been employed, with and among their dangerous tools, and, —Art's noblest trophy,—the restoration of reason and intellect ensued."

The Report of the committee of visitors of the *Bedford Lunatic Asylum* for 1847, contains nothing which entitles it to special notice. The number of patients in the asylum at the close of 1846, was 289; admitted during the year, 101. The daily average number of patients during the year has been 190. The receipts for the year have been £3,911 7s., and the expenditure £3,945 13s. 7d. A balance of £117 16s. 10d. was due to the treasurer on the 31st December, 1846, and £152 3s. 5d. at the end of the last year.

The medical section of the Report, drawn up by Mr. Harris, the visiting-surgeon, and superintendent of the establishment, contains some suggestions with reference to the internal management of the asylum, which we trust the "committee of visitors" will well consider. It appears that influenza has prevailed to some extent in the asylum, but the cases have been more tractable than formerly, though some have degenerated into the low typhoid fever that has prevailed to such an extent in the neighbourhood.

Erysipelas has made its appearance in several patients, but it has not been followed by serious consequence in any case. No other disease has appeared in a greater ratio than might be expected.

One case of suicide, in a young female, has unfortunately occurred; and Mr. Harris expresses his surprise that such events are not more frequent, when we consider the great number of patients that have been admitted prone to self-destruction. With all the care and watchfulness that can be exercised, it is impossible to guard against such accidents entirely.

A man who was not suspected of a suicidal tendency, after getting up and coming down stairs, went up again and suspended himself by his handkerchief, but he was fortunately cut down ere it was too late. This man did not attempt suicide from a wish to die, nor to get rid of trouble, but because he was "ordered to do it" by the "spirits"—by the "higher powers;" and he told Mr. Harris that if he received such "strong orders" again, he should repeat the attempt.

Mr. Harris complains of a *want of offices.* The asylum was originally built for forty patients. It now contains *one hundred and ninety!* The increase in the number of patients' rooms has not been accompanied by an increase in the number of offices. He also complains of an insufficient number of servants, and that they are indifferently paid. Surely these are important points for consideration. We trust that the next Report will be more satisfactory in its character. The committee of visitors may rest assured that nothing is gained in the management of the insane by niggard economy—that no philosophical system of medical and moral treatment can be carried out without the medical officer has the co-operation of a sufficient number of efficient servants, and that good, trustworthy attendants or servants cannot be obtained unless they are liberally remunerated for the discharge of the onerous duties that devolve upon them.

The following statistical facts, published in the Report of the Medical Officer (Dr. J. E. Huxley) and Superintendent of the *Kent Lunatic Asylum,* for 1847, will be considered interesting:—

| | |
|---|---|
| Remaining in the asylum 4th July, 1846 | 324 |
| Admitted in the year following | 108 |
| Number under treatment during the year | 432 |
| Deduct, number discharged in the year | 86 |
| Number in asylum July 4th, 1847 | 346 |
| Total number of admissions from opening to July 4th, 1847. Males, 552; Females, 476 | 1028 |
| Total number discharged—Males, 406; Females, 276 | 682 |
| Remainder (as above) | 346 |

Of the number under treatment during the year, there have been discharged—

| | |
|---|---|
| Cured—Males, 17; females, 14—31 <br> On trial since reported cured—Females, 5 | 36 |

or $33\frac{1}{3}$ per cent. on the admissions, and nearly 10 per cent. increase on the rate for all the preceding years.

| | | |
|---|---|---|
| Removed, at desire of friends | 1 | |
| „ to another asylum | 1 | 4 |
| „ harmless and further incurable | 2 | |
| Escaped (a male) | | 1 |
| Died—Males, 26; Females, 19 | | 45 |
| Total disposed of | | 86 |

| | |
|---|---|
| Number admitted during the year | 108 |
| Do. disposed of | 86 |
| Excess of admissions over discharges | 22 |

or nearly 7 per cent. increase in the number accommodated.

Of the deaths, 45 in number, 41 occurred in incurable cases, as regards the description and duration of the mental disease. The remaining 4 were destroyed by disorders, not necessarily accompanying, although commonly incidental to, insanity.

*Of the* 45 *who died:*

| | | |
|---|---|---|
| 11 | had been in the asylum from | 10 to 14 years. |
| 2 | „ „ | 8 to 9 „ |
| 18 | „ „ | 1 to 4 „ |
| 14 | „ „ | under 1 year. |

Total 45, or 41 per cent. on the admissions of the year, being 11 per cent. in excess of the rate for all preceding years, and a very large mortality, explicable, however, by reference to the advanced age of a large number of the deceased, and the many occurrences of complication of serious bodily disease, and the severity of the past winter.

Regarding the inmates of the asylum during the year, as the inhabitants of a village, for the sake of a comparison of mortality, the rate will be found to be about 10 and 4-tenths per cent.

*Ages of those who died:—*

| | Males. | Females. | Total. |
|---|---|---|---|
| 80 years and upwards | 1 | 1 | 2 |
| 70 to 80 | 0 | 3 | 3 |
| 60 to 70 | 3 | 1 | 4 |
| 50 to 60 | 4 | 3 | 7 |
| 40 to 50 | 5 | 2 | 7 |
| 20 to 40 | 13 | 7 | 20 |
| under 20 | 0 | 2 | 2 |
| | 26 | 19 | 45 |

Fourteen died with paralysis, a fatal complication, and of these, four had been admitted during the year; nine were epileptics, and six of these died in direct consequence of the frequency and severity of their fits; six were congenital idiots, two of these being also epileptics. All the deaths occurred in cases of mental disorder of the least hopeful forms; namely, dementia, or decay of the mental powers, and idiocy; with the exception only of five cases (four of mania, one of melancholia) which were all complicated with and determined by severe structural diseases of the brain, heart, or lungs.

*Tabular view of the causes of death.*

| | Males. | | Females. | | Total. |
|---|---|---|---|---|---|
| Exhaustion (maniacal) | 0 | .. | 3 | .. | 3 |
| „ (from abscess) | 1 | .. | 0 | .. | 1 |
| „ (with diseases of lungs, &c.) | 5 | .. | 3 | .. | 8 |
| „ (simple, in-patients of advanced age) | 5 | .. | 5 | .. | 10 |
| Paralysis | 8 | .. | 5 | .. | 13 |
| Apoplexy (independent on epilepsy) | 1 | .. | 0 | .. | 1 |
| Epilepsy | 2 | .. | 3 | .. | 5 |
| „ (with paralysis) | 1 | .. | 0 | .. | 1 |
| Disease of Heart | 1 | .. | 0 | .. | 1 |
| „ Brain (abscess) | 1 | .. | 0 | .. | 1 |
| „ „ and Lungs | 1 | .. | 0 | .. | 1 |
| | 26 | | 19 | | 45 |

*Time in the Asylum of all the Patients that are therein.*

| | |
|---|---|
| Confined above 14 years | 20 |
| „ 13 to 14 „ | 19 |
| „ 12 to 13 „ | 17 |
| „ 10 to 12 „ | 17 |
| „ 8 to 10 „ | 20 |
| „ 6 to 8 „ | 22—or 115 who have been from 6 to 14 years in confinement, or still remaining out of the admission of the first 8 years; |
| Confined 4 to 6 years | 47 |
| „ 2 to 4 „ | 52 |
| „ 1 to 2 „ | 54 |
| „ 1 year and under | 78—Total, 346. |

*Fit for removal to the Chronic House.*

Males ...... 50; Females ...... 71; Total ...... 121

The Reports for 1846 and 1847 of the *Suffolk Lunatic Asylum* contain many interesting particulars. The admissions, discharges, and deaths are as follows:—

| | Males. | | Females. | | Total. |
|---|---|---|---|---|---|
| The numbers admitted this year | 37 | .... | 44 | .... | 81 |
| Discharged | 19 | .... | 25 | .... | 44 |
| Died | 16 | .... | 15 | .... | 31 |
| In the House this day | 114 | .... | 131 | .... | 246* |

The following case will interest our readers—

"E. J., a casual patient, was found in an adjacent parish, to which she came by the coach from London, without being able to give any collected statement of her previous history, or former place of abode. Her discourse was generally incoherent, and she appeared the subject of decided mania. She was taken care of for a few days at a small inn, where it appears she needed daily and nightly watching, to prevent self-destruction. On her admission (on Sept. 10th) she talked of going to Calcutta and home again, in twenty-four hours; and, with an exhibition of insane pride, declared she was related to, and the representative of several noble houses, and the holder of large Indian possessions. The Calcutta journey, in a few days after her admission,

* The Cures are *above*, the Deaths *about* the average on general numbers.

was abandoned for a more important embassy to Damascus, and this prevailing monomania continued for some days. She was an interesting, well-informed woman, very tractable, and would readily adopt any means suggested for improvement in her bodily health, and listen to arguments on her mental condition. She continued under general treatment, medical and moral, for about two months, throughout which period she progressively improved. Her health became re-established, and her delusions left her. Her true history was then ascertained, and found to be, as she stated herself, a short time before she went away, a very distressing one. She had been a confidential servant in a nobleman's family, where she lived much respected, and with great comfort, for *eleven* years. She saved some money in her employ as housekeeper, and married. Circumstances occurred in her domestic history, of a more than ordinarily painful character, which led to her miscarriage at five months, and she has been since (between five and six years) the subject of recurrent mania. Upon any sudden anxiety, or unlooked for perplexity, she says 'her knees strike together, and a noise strikes up in her head.' All her attacks are attended with, and frequently *preceded by* strong suicidal inclinations. Her own words are, 'I have often felt on being seized with insanity, an immediate, and quite irresistible impulse to commit self-destruction, from which I have been wonderfully preserved. All my prayer when I *have* my reason is, that I may be kept from a successful attempt. I was once so distressed from this cause alone, that I applied to a minister of the gospel, and asked him, 'Do you think that I am accountable for deeds done in the dark hour of insanity?' Of course this minister of peace assured her of the contrary, and thus successfully combated a fearful feeling, and treated this case of insanity well.

"During the latter period of her residence here, she was always actively and cheerfully employed, working very beautifully at a superior kind of worsted work, and instructing others. She was discharged perfectly well in November, and left, with an attendant, for London on the 11th, expressing herself as 'happy at going, but grieved to go.'"

The following data convey an idea of the admissions, &c., in 1847—

| | Males. | Females | Total. |
|---|---|---|---|
| Patients in the House Dec. 31st, 1846.... | 114 | 131 | 245 |
| Admitted in 1847 ............ | 42 | 42 | 84 |
| | 156 | 173 | 329 |
| Patients Discharged,—cured ............ | 16 | 15 | 31 |
| ,, ,, Relieved.......... | 1 | 0 | 1 |
| Died ...................... | 24 | 24 | 48 |
| Escaped .................... | 1 | 0 | 1 |
| | 42 | 39 | 81 |
| In the House this day, Dec. 17th, 1847 .. | 114 | 134 | 248 |

Dr. Kirkman observes—

"In order to carry out the steady pursuit of any course to a successful issue, there are lessons which can only be learned in the Insane School; and even there it is sometimes difficult to tutor one's own mind to the conviction, that the doings of turbulent destructiveness are beyond individual control; and that that class of patients who come under the distinction of 'Morally Insane,' are quite irresponsible for their deeds. The moment, however, responsibility is admitted, penal treatment is the necessary sequence, which never should arise in the moral management of the insane, or enter into the discipline of a lunatic asylum. There are cases, however, which are occasionally met with, that call for more than ordinary forbearance, and, in the zeal for representative quietude, it is hardly honest not to notice them. We have an anxious case in the house at present, of a female whose discourse is always on the 'outrage' (to use her own words) to which her 'fine feelings are subjected by association with lunatics,' while her daily and nightly displays are themselves coarse and outrageous. The number of windows this woman has broken, and the blankets she has torn, would make up a very serious amount of expenditure. Her protest against her present associations, is perhaps not altogether without some colour. She was brought up in respectable life, but, from having shown of late years symptoms of insanity, was removed from her own cottage and placed in a private asylum; from there, without having returned home, or being herself at all convinced of her altered pecuniary circumstances, she was brought to this house as a pauper; where she still is a tax upon our ingenuity to devise means in prevention of the exercise of her destructive tendency. There is little doubt that her mania has been aggravated by deceptions practised; and she must be added to the list of those alluded to in the last Report, suffering from injudicious, though perhaps not ill-intentioned conduct. She has a great dislike to visitors, keeps in her room as much as possible, and is always excited by strangers She is now the subject for decision on that nice point between that inspection and publicity, which should *always be;* and those visits for mere gratification and curiosity, which should *never be*."

These cases are, alas! of frequent occurrence. They are to be found frequently in the bosom of private families, the insanity of the parties remaining unnoticed until some fearful explosion forces a conviction of truth on unwilling minds.

The Physicians' Report of the *Royal Hospital of Bethlem*, for 1847, drawn up by Sir A. Morison, M.D., and Dr. Edward Thomas Monro, contains many important facts. During the year 1846, 293 patients were admitted, of whom 125 were males, and 168 females; 160 patients had been discharged cured, consisting of 66 males, and 95 females; being 56 per cent. The uncured consist of 87. The number of deaths was 15. With regard to the general health of the patients the Report is satisfactory. It is gratifying to hear that the average attendance of patients, both male and female, at divine service, has amounted to $132\frac{4}{27}$, and that no instances of impropriety on the part of the patients have been manifested. The advocates of what is termed the "non-restraint system of treatment" will be pleased to hear that the degree of restraint to which the patients during 1846 were subjected, has been almost nominal. The ratio of

| | | | |
|---|---|---|---|
| 1840, was . . . . . . . | $13\frac{20}{52}$ | 1843, was . . . . . . . | $3\frac{4}{52}$ |
| 1841, „ . . . . . . . | 9 | 1844, „ . . . . . . . | $1\frac{35}{52}$ |
| 1842, „ . . . . . . . | 3 | 1845, „ . . . . . . . | $1\frac{20}{52}$ |

That of last year stands at two-thirds of one.

No accident of any moment has occurred, no suicide, or event demanding an inquest; which, considering the large number of patients, and the strong tendency of many to injure themselves, is a matter of congratulation, and speaks volumes as to the care and vigilance exercised by the nurses and attendants. The padded rooms have been used occasionally, and are found very generally useful at night, as well as in those cases which now and then arise by day.

With regard to the incurable patients, the Report says—

"On the 31st of December the number of patients of this class was, including those on leave of absence, eighty-four, consisting of forty-four females and forty males, being five fewer than at the close of the preceding year.

"During the year there have been admitted three males; the same number of males have died, and five females. Only four patients now remain of those who, in 1815, were transplanted from the old hospital in Moorfields, being two of either sex, of whom an aged female of eighty-four years is still what may be justly termed the mother of the hospital, and enjoys all her faculties, though still afflicted with a sort of religious depression, and much disabled by rheumatism. Her calm and kindly manners are the admiration of all."

On the subject of the criminal lunatics it appears—

"There have been admitted during the present year fourteen males and three females; and there has died one female; while one of either sex has been discharged well, under the warrant of the Secretary of State.

"There remain 111, consisting of ninety males and twenty one females.

"The patients in this department are healthy, and generally speaking cheerful and active. One patient was attacked by erysipelas of a formidable character, but who ultimately entirely recovered, and continues in good health."

Admitted during the year 1846—

| | Males. | Females. | Total. |
|---|---|---|---|
| Curables | 200 | 303 | 503 |
| Incurables | 43 | 49 | 92 |
| Criminals | 91 | 23 | 114 |

The average number of patients daily employed has been 251; 127 males, and 124 females.

"Total number of curable patients admitted into Bethlem Hospital during 100 years, ending the 31st December, 1843, with the amount of cures and deaths.

| | |
|---|---|
| Total patients admitted | 17·803 |
| Discharged cured | 7108, or 39·86 per cent. |
| Died | 1799, or 10·10 per cent. |

It appears from a table exhibiting the monthly admissions and cures of patients during the year 1846, that the greatest number of patients were admitted during the months of June, July, and October. It also would appear that the ages most liable to insanity, taking as data the 293 curable patients who were admitted during 1846, were from twenty to twenty-five, thirty to thirty-five, and from thirty-five to forty. Most of the curable patients were admitted at an early period of the attack. The largest number of patients (thirty-eight) had been ill for only two weeks. One hundred and ninety-seven patients entered the Hospital on their first attack. With regard to the social position of the patients, there were

| | |
|---|---|
| Married | 145 |
| Single | 126 |
| Widowed | 22 |

Of the whole admissions of curable patients, 171 were returned as indifferently educated, fifteen had no education at all. The provinces appear to have supplied the greater number of patients (174); the metropolis, 95. Two hundred and three of the patients belonged to the Church of England; Independents, 20; Wesleyans, 17; Roman Catholics, 12; Baptists, 11. Among the same class of patients (curable) there were—

| | |
|---|---|
| Dangerous patients | 98 |
| Violent ditto | 61 |
| Neither | 159 |

Thirty-four patients had attempted suicide prior to admission; sixty had meditated self-destruction.

*Apparent and Assigned Causes of Disease in Patients discharged Cured.*

### MALES.

### MORAL.

| | |
|---|---|
| Religion | 4 |
| Anxiety | 4 |
| Domestic troubles | 2 |
| Loss of situation | 2 |
| Want of employment | 2 |
| Over study | 1 |
| Disappointment | 1 |
| Over anxiety in business | 2 |
| Embarrassment | 1 |
| Losses in business | 1 |
| Over exertion in business | 2 |
| Over study of religious subjects | 1 |
| Ill treatment of a servant | 1 |
| Anxiety about losses | 1 |
| Disappointed affection | 1 |
| Embarrassed circumstances | 1 |
| | 27 |

### PHYSICAL.

| | |
|---|---|
| Intemperance | 7 |
| Excessive heat of the weather | 2 |
| Over exertion | 2 |
| Injury of the head | 1 |
| Sensual excess | 3 |
| Fall from a horse | 1 |
| Blow on the head | 2 |
| | 18 |

### HEREDITARY.

| | |
|---|---|
| Hereditary tendency to insanity was traced in 21 cases, of which 12 appeared to be without any other obvious cause | 12 |
| Not ascertained | 9 |
| Total | 66 |

### FEMALES.

### MORAL.

| | |
|---|---|
| Love | 4 |
| Anxiety | 4 |
| Religion | 2 |
| Fright | 3 |
| Death of relatives | 4 |
| Disappointed affection | 4 |
| Desertion by her husband | 1 |
| Change of residence | 3 |
| Sudden change from quiet to bustle | 1 |
| Over study of religious subjects | 2 |
| Death of husband | 1 |
| Embarrassed circumstances | 1 |
| Death of child | 1 |
| | 31 |

### PHYSICAL.

| | |
|---|---|
| Puerperal | 10 |
| Uterine disturbance | 4 |
| Intemperance | 3 |
| Change of life | 2 |
| Protracted lactation | 5 |
| Over exertion while suckling | 1 |
| Cessation of tic do. | 1 |
| Nervous disease | 1 |
| Bodily illness | 1 |
| Typhus fever | 1 |
| Scarlet fever | 1 |
| Frequent attacks of diarrhœa | 1 |
| | 31 |

### HEREDITARY.

| | |
|---|---|
| Hereditary tendency to insanity was traced in 33 cases, of which there appeared to be 20 without any other obvious cause | 20 |
| Not ascertained | 13 |
| Total | 95 |

*Synopsis of Offences of the Criminal Lunatics confined in Bethlem Hospital,* 31st December, 1846.

| NATURE OF OFFENCE. | | Males. | Females. | Total. |
|---|---|---|---|---|
| 1. Against the State | M. | | | |
| (1.) High Treason | 1 | | | |
| (2.) Sedition | 1 | | | |
| | 2 | 2 | — | 2 |
| 2. Against the Person | | 57 | 13 | 70 |
| 3. Against Property | | 31 | 8 | 39 |
| | | 90 | 21 | 111 |

*Time the Criminal Lunatics have been in Bethlem Hospital.*

| | Males. | Females. | Total. |
|---|---|---|---|
| Not exceeding 30 years ................ | 4 | 1 | 5 |
| " 28 " ................ | 7 | 1 | 8 |
| " 25 " ................ | 2 | 3 | 5 |
| " 20 " ................ | 3 | .. | 3 |
| " 15 " ................ | 1 | .. | 1 |
| " 10 " ................ | 21 | 4 | 25 |
| " 5 " ................ | 10 | 3 | 13 |
| " 3 " ................ | 28 | 6 | 34 |
| " 1 " ................ | 14 | 3 | 17 |
| | 90 | 21 | 111 |

We have been favoured with the 51st Report of the *Friends' Retreat* near York, for 1847, by which it appears that the number of patients in the house is 113—viz., 44 men and 69 women, of whom fifteen are wholly unconnected with the Society of Friends. The income during 1846, was 5487*l.* 2*s.* 3*d.*, 4983*l.* 19*s.* 6*d.* of which was received from patients. The expenditure was 4487*l.* 2*s.* 3*d.*. The property belonging to the institution amounts to 23,216*l.* 4*s.* 8*d.* Owing to the institution, 23,216*l.* 4*s.* 8*d.* The donations for the year 1846 were only 46*l.*; annual subscriptions, 322*l.* The housekeeping expenses were 2544*l.* 3*s.* 6*d.* The expenditure for the principal articles in housekeeping was as follows :—

| | £ | s. | d. |
|---|---|---|---|
| Butchers' Meat and Bacon, 2199st., at 6s. 7½d. ......... | 726 | 5 | 4 |
| Milk, 6366 gallons, at 8d. ............................ | 212 | 4 | 2 |
| Butter, 3328lb., Lard and Eggs ...................... | 218 | 12 | 11 |
| Flour, 140 sacks, at £2 10s. 9d. ...................... | 355 | 6 | 10 |
| Tea and Coffee ........................................ | 169 | 0 | 0 |
| Sugar .................................................... | 165 | 0 | 0 |
| Fruit and Vegetables .................................. | 157 | 0 | 0 |
| Coals .................................................... | 165 | 0 | 0 |

The family consisted during the year on an average of 145 persons, exclusive of visitors. The farm and garden yielded 719*l.* 4*s.* 2*d.* Patients admitted during the year, 23; of these admitted for the first time, there were 14; re-admissions, 9. Eighteen patients were discharged during the year; recovered, 11; improved, 1. The admissions, re-admissions, discharges, and deaths during the last 50 years in the Retreat, between the years 1796 and 1847, were as follows :—

| | Males. | | Females. | | Total. |
|---|---|---|---|---|---|
| Admissions ...... | 274 | .... | 301 | .... | 575 |
| Re-admissions .... | 82 | .... | 110 | .... | 192 |
| | 356 | .... | 411 | .... | 767 |

Discharged recovered, 362 patients—158 males and 204 females; improved, 79; unimproved, 40; died, 173. Total discharged and died during the 51 years, 654; of these, 312 were male, and 342 female patients. The following tabular statements will deeply interest our readers :—

TABLE IV.—*Showing the Average Proportion of Recoveries and the Mean Annual Mortality, in Cases of Recent and Longer Duration when Admitted,* 1796—1847.

| DURATION OF DISORDER WHEN ADMITTED. | Proportions of Recoveries per cent. of the Admissions. | | | Mean Annual Mortality per cent. Resident. | | |
|---|---|---|---|---|---|---|
| | Male. | Female. | Mean. | Male. | Female. | Mean. |
| FIRST CLASS.—First attack, and within three months .......... | 78·57 | 76·27 | 77·39 | 9·21 | 6·08 | 7·32 |
| SECOND CLASS.—First attack, above three, within twelve months.... | 48·14 | 43·13 | 45·71 | 5·16 | 4·05 | 4·38 |
| THIRD CLASS.—Not first attack, and within twelve months .... | 58·33 | 65·43 | 62·41 | 5·91 | 4·64 | 5·04 |
| FOURTH CLASS.—First or not first attack, and more than twelve months .................... | 14.28 | 22·30 | 18·49 | 5·28 | 3·92 | 4·55 |
| Average ............ | 44.38 | 49·63 | 47·19 | 5·58 | 4·16 | 4·76 |
| AVERAGE, EXCLUSIVE OF THOSE UNCONNECTED WITH THE SOCIETY OF FRIENDS ................ | 45 8 | 52·56 | 49·24 | 5·45 | 4 89 | 4·67 |

TABLE V.—*Showing the Ages of those Admitted at the Time of the First Attack, and upon Admission; with the Mean Numbers Resident at Decennial Periods of Life, during the Year* 1846—47.

| AGE. | Age at First Attack.* | | | Age at Admission and Re-admission. | | | Mean Numbers Resident at Decennial periods of Life. | | |
|---|---|---|---|---|---|---|---|---|---|
| | Male. | Fem. | Total. | Male. | Fem. | Total. | Male. | Female. | Total. |
| From 10 to 15 years. | 1 | .. | 1 | .. | .. | .. | .. | .. | .. |
| " 15 " 20 " | .. | 1 | 1 | .. | 1 | 1 | .. | ·91 | ·91 |
| " 20 " 30 " | 2 | 4 | 6 | 4 | 5 | 9 | 4 58 | 7·92 | 12·50 |
| " 30 " 40 " | 2 | 1 | 3 | 4 | 1 | 5 | 13·84 | 14· | 27·84 |
| " 40 " 50 " | .. | 1 | 1 | .. | 1 | 1 | 5· | 14·25 | 19 25 |
| " 50 " 60 " | 1 | .. | 1 | 1 | 1 | 2 | 12·41 | 7·75 | 20·16 |
| " 60 " 70 " | .. | 1 | 1 | 2 | 3 | 5 | 4·67 | 14·17 | 18·84 |
| " 70 " 80 " | .. | .. | .. | .. | .. | .. | 1·75 | 7·33 | 9·08 |
| " 80 " 90 " | .. | .. | .. | .. | .. | .. | .. | 1·58 | 1·58 |
| Total .... | 6 | 8 | 14 | 11 | 12 | 23 | 42·25 | 67·91 | 110·16 |
| | Male. | Fem. | Mean. | Male. | Fem. | Mean. | Male. | Female. | Mean. |
| AVERAGE AGES FOR 44 YEARS, 1796 —1840, IN YEARS | 33·5 | 35·1 | 34·4 | 38·3 | 39·9 | 39·2 | 48·5 | 49·1 | 48·9 |

* The age at first attack of the nine re-admitted cases having been given in previous years, is not repeated here.

